# Osteogenic parameters surrounding trabecular tantalum metal implants in osteotomies prepared via osseodensification drilling

**DOI:** 10.4317/medoral.23108

**Published:** 2019-10-27

**Authors:** Lukasz Witek, Adham M. Alifarag, Nick Tovar, Christopher D. Lopez, Luiz F. Gil, Michelle Gorbonosov, Kaitlin Hannan, Rodrigo Neiva, Paulo G. Coelho

**Affiliations:** 1 Department of Biomaterials and Biomimetics, New York University College of Dentistry, New York, NY; 2 College of Medicine, SUNY Upstate Medical University, Syracuse, N; 3 Icahn School of Medicine at Mount Sinai, New York, NY; 4 Department of Periodontics, School of Dental Medicine, University of Pennsylvania, Philadelphia, PA; 5 Department of Morphological Sciences, Federal University of Santa Catarina (UFSC), Florianópolis, Santa Catarina, Brazil; 6 Hansjörg Wyss Department of Plastic Surgery, New York University Langone Medical Center, New York, NY

## Abstract

**Background:**

Surgical fixation of implants into bone for the correction of bone deformities or defects is a traditional approach for skeletal stabilization. Important measures of efficacy of implants include implant stability and osseointegration—the direct interaction between living bone and an implant. Osseointegration depends on successful implant placement and subsequent bone remodeling. This study utilized osseodensification drilling (OD) in a low bone density model using trabecular metal (TM) implants.

**Material and Methods:**

Three osteotomy sites, Regular, OD-CW (clockwise), and OD-CCW (counterclockwise), were prepared in each ilium of three female sheep. Drilling was performed at 1100rpm with saline irrigation. Trabecular metal (TM) (Zimmer®, Parsippany, NJ, USA) implants measuring 3.7mm in diameter x 10mm length were placed into respective osteotomies. A three-week period post-surgery was given to allow for healing to take place after which all three sheep were euthanized and the ilia were collected. Samples were prepared, qualitatively and quantitatively analyzed using histology micrographs and image analysis software (ImageJ, NIH, Bethesda, MD). Bone-to-implant contact (BIC) and bone area fraction occupancy (BAFO) were quantified to evaluate the osseointegration parameters.

**Results:**

All implants exhibit successful bone formation in the peri-implant environment as well as within the open spaces of the trabecular network. Osseointegration within the TM (quantified by %BIC) as a function of drilling technique was more pronounced in OD samples(*p*>0.05). The %BAFO however shows a significant difference (*p*=0.036) between the CCW and R samples. Greater bone volume and frequency of bone chips are observed in OD samples.

**Conclusions:**

The utilization of OD as a design for improved fixation of hardware was supported by increased levels of stability, both primary and secondary. Histological data with OD provided notably different results from those of the regular drilling method.

** Key words:**Osseodensification drilling, trabecular tantalum metal, osteotomies, implants, subtractive drilling.

## Introduction

Successful integration of an implant with bone may be influenced by factors such as surgical protocol (presence of irrigation, speed (RPM), drilling sequence, etc.), device geometrical conFigurations, or surface chemistry modifications (e.g. chemical coating such as calcium phosphate (CaP) crystals or hydroxyapatite) (1,2). A common goal among treatment protocols is osseointegration, the direct structural and functional connection between living bone and the implanted device. Osseointegration is an essential component in ensuring long-term stability, which if compromised, may lead to complications that often result in the need for a second surgical procedure (3). Additional surgical interventions are associated with an increased financial burden on the patient, higher risk of infection, and surgical site morbidity (4,5).

The number of revision surgeries is expected to increase if strategies for long-lasting implant fixation are not put into place (5). Readmission rates for hip arthroplasty and total knee replacement surgeries are expected to increase by 137% and 601%, respectively, over the next 25 years (6). Successful osseointegration significantly minimizes the probability of implant failure in the long term (7), and thus focusing on improving osseointegration is a priority. For osseointegration to take place during the healing period, the implant needs to be in direct contact with, or in close proximity to, an adequate volume of bone which is usually indicated by a strong primary stability (8). Primary stability is usually measured via insertion torque, where higher values indicate a more rigid fixation into bone due to increased bone density in the peri-implant environment (9).

Although numerous techniques have been designed or tailored to promote increased primary stability, some of the techniques still face limitations, which can potentially limit the device’s ability to attain primary stability, or osseointegration (2). For example, the use of hydroxyapatite (HA)-coated implants was implemented to take advantage of the osteoconductive nature of the mineralized matrix, but HA has been subject to rapid wear which can diminish the implant’s osseointegrative potential (2,10). Another implant modification, which has an influence on osseointegration is the surface roughness/texture of the implant (11). Porous implants have been shown to osseointegrate better with surrounding bone compared to implants with a smooth surface (i.e., as machined) as the degree of roughness dictates the surface energy which contributes to osteogenic protein adsorption, cell adhesion, and cell proliferation.(12,13). In addition, the increased surface area is met with an increased amount of bone in contact with the implant.

An additional variable is in the preparation of the osteotomy is the use of irrigation. Irrigation while drilling provides lubrication and cools the bur and contact surfaces being drilled which prevents overheating of the bone, in essence preventing osteonecrosis followed by extensive osteoclastic activity (14-16). An increased level of osteoclastic activity can diminish the bone volume surrounding the implant’s threads which has potential to lead to an adverse effect with respect to osseointegration. The predicament in some studies associated with irrigation, that copious amounts of irrigation there is a potential that essential osteogenic signaling proteins, which are normally be located within the osteotomy, or osteoconductive bone fragments can be ‘washed’ away (17). More recently studies have focused on modifying the drilling protocol to better promote primary stability and osseointegration (17-20).

The most common process, conventional drilling, which is subtractive in nature, results in the excavation of bone fragments that would typically act as nucleating surfaces for osteoblastic activity (2,8). A newer, more modern, technique, termed osseodensification, uses an innovative bur, designed to allow for additive drilling (2,8,21). Bone fragments created during drilling are displaced laterally and result in densification of the osteotomy wall via osteocompaction (2,8,21). The bone fragments have shown to significantly increase primary stability (21), while simultaneously functioning to bridge the gap created between the implant surface and osteotomy wall.

The purpose of this study was to quantitively and qualitatively assess the effect of osseodensification drilling on the highly porous (~80%) implant with a trabecular metal morphological component in low density bone environment.

Materials and Methods 

- Preclinical laboratory *in vivo* model

**Figure 1:**
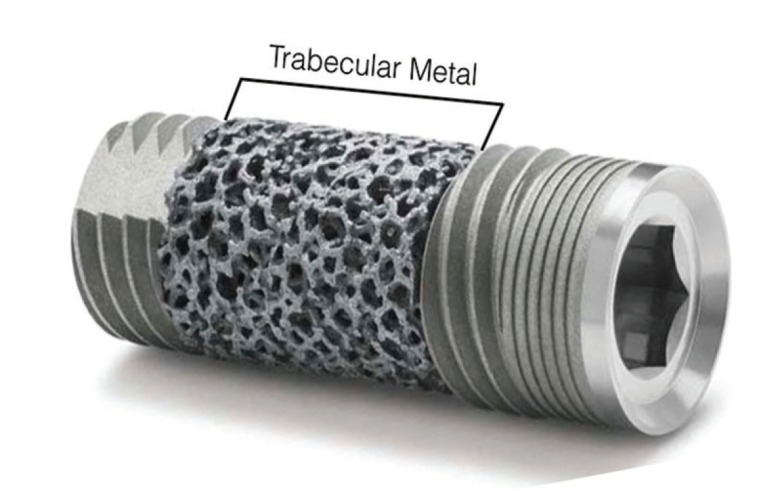
Digital image of the trabecular metal (TM) implant and its morphology.

Upon receiving approval from the Institutional Animal Care and Use Committee (Comité d’éthique Anses/ENVA/UPEC Approval Reference#: 13-011) three female sheep (each weighing ~65 kg) were acquired and allowed to acclimate for ~5 days. Trabecular metal (TM) (Zimmer®, Parsippany, NJ, USA) implants were utilized, with dimensions of 3.7-mm in diameter and 10-mm in length (Fig. 1).

Prior to surgery, anesthesia was induced with sodium pentothal (15-20mg/kg) in Normasol solution into the jugular vein and maintained with isoflurane (1.5-3%) in O2/N2O (50/50). Animal monitoring included ECG, end tidal CO2, and SpO2 and body temperature, which was regulated by a circulating hot water blanket. Prior to surgery, the surgical sites (bilateral hip) were shaved and iodine solution was applied to prepare surgical site. A ~10 cm incision was placed along the iliac crest, dissections of fat tissue were performed and muscular tissue was reached. Dissection of muscular plane was performed with blunt dissection and the ilium was exposed using a periosteal elevator.

Three osteotomy sites were prepared in each of the ilia, (regular [R] (subtractive), clockwise [OD-CW], and counterclockwise [OD-CCW]). TM implants was subsequently placed in R osteotomy sites prepared using a 3-step regular surgical drilling technique of 2.0 mm pilot, 2.8 mm and 3.4 mm twist drills as recommended by the implant manufacturer. OD-CW and OD-CCW drilling sites were subjected to osseodensification (OD) (additive) drilling using the Densah Bur (Versah, Jackson, MI, USA) 1.7 mm pilot, 2.8 mm, and 3.8 mm multi fluted tapered burs. TM implants were then placed in the CW and CCW osteotomy sites. All drilling techniques were performed at 1100 rpm and with saline irrigation.

The surgical site was closed with a layered technique using Vicryl 2-0 for muscle and 2-0 nylon for skin. Cefazolin (500 mg) was administered intravenously pre-operatively and post-operatively. Post-operatively, food and water *ad libitum* was offered to the animals.

All sheep were euthanized by anesthesia overdose at three weeks post-surgery. Upon sacrifice, the hips were collected by sharp dissection. All samples were referred for histological processing.

- Histological preparation and histomorphometry

Implants along with surrounding bone tissue were removed *en bloc* for non-decalcified histological processing. The bone-implant blocks were gradually dehydrated in a series (70-100%) of ethanol solutions and then embedded in a methyl methacrylate-based resin. Embedded blocks were then cut into sections using a diamond saw (Isomet 2000, Buehler Ltd., Lake Bluff, IL, USA). The sections were glued to slides and ground on a grinding machine (Metaserv 3000, Buehler, Lake Bluff, IL, USA) under water irrigation with a series of SiC abrasive paper (Buehler, Lake Bluff, IL, USA) until they were approximately 100 μm thick. The samples were then stained in Stevenel's blue and Van Geison to differentiate the soft and connective tissues.

Samples were qualitatively and quantitatively analyzed using histology micrographs and image analysis software (ImageJ, NIH, Bethesda, MD). Bone-to-implant contact (BIC) and bone area fraction occupancy (BAFO) were quantified to evaluate the osseointegration parameters of the trabecular metal portion of the implant (Fig. 1). BIC determines the degree of osseointegration by tabulating the percentage of bone contact over the entire relevant implant surface perimeter while BAFO quantifies bone growth within the implant threads as a percentage (22,23).

- Statistical analysis

All histomorphometric testing data are presented as mean values with the corresponding 95% confidence interval values (mean ± CI). %BIC, and %BAFO data were analyzed using a linear mixed model with a fixed factor of surgical drilling method: Regular (R), clockwise (OD-CW), and counterclockwise (OD-CCW). All analyses were completed with IBM SPSS (v23, IBM Corp., Armonk, NY).

## Results

No surgical site showed signs of inflammation or infection during immediate post-operative evaluation. No evident failure of implants was observed at time of necropsy.

- Histomorphometric Analysis 

**Figure 2:**
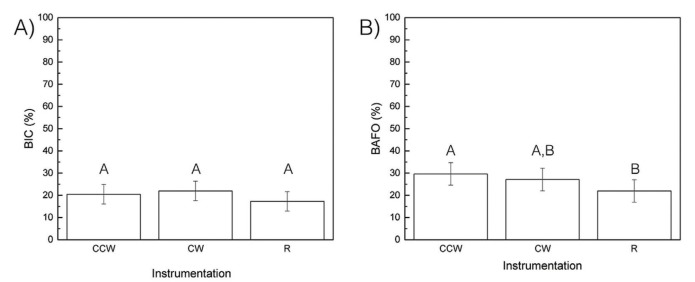
Histomorphometric data. (a) BIC and (b) BAFO as a function of surgical technique. The letters indicate statistically homogenous groups.

Analyzing the level of integration within the trabecular metal portion of the implant as a function of drilling technique showed an, although not statistically different, increasing trend, of %BIC in samples drilled with OD relative to samples prepared through the conventional R drilling instrumentation (Fig. 2) (*p*>0.05).

While the %BAFO however shows a significant difference (*p*=0.036) between the CCW and R samples. Additionally, a pairwise analyses between CCW-CW and CW-R resulted in statistically homogenous values (Fig. 2).

- Histological analysis

**Figure 3:**
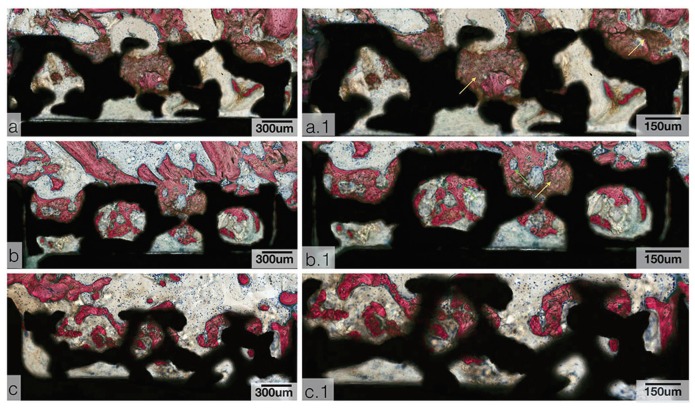
Survey histological micrographs for TM implants. (a) CCW, (b) CW, (c) R. Samples stained with Van Geison’s fuchsin and Stevenel’s blue. With high magnification histological micrographs of TM implant samples. (a.1) CCW, (b.1) CW, and (c.1) R. Yellow arrows depict bone chip residues, and green arrows depict bone remodeling sites. Samples stained with Van Geison’s fuchsin and Stevenel’s blue.

Qualitative histologic evaluation indicated that all implants exhibit successful bone formation in the peri-implant environment as well as within the open spaces of the trabecular network (Fig. 3). Compared to samples instrumented via R drilling, the bone volume in samples drilled with OD is more pronounced (Fig. 3). CCW samples show bone chips within the TM and in the proximity as well (Fig. 3), whereas the presence of these chips is seldom seen in CW and R samples (Fig. 3).

During instrumentation, bone chips caught in the TM acted as nucleating sites for osteogenesis. The presence of woven bone in the TM suggests successful bony ingrowth and vascularization throughout the porous network.

## Discussion

Osseointegration is crucial for the success of implant placement into bone. Successful fixation avoids the need for revision surgery which is often required in the event of implant failure, e.g. loosening from the bone socket, or implant fracture. These revision surgeries are accompanied with higher healthcare costs, increased risk of infection, and surgical site morbidity. Osseodensification is a novel approach to osteotomy preparation for implant placement that has shown to improve parameters such as primary stability, osseointegration, and secondary stability—all indicators of long-term implant survival from the time of fixation to the healing period. This study sought to further evaluate the effects of osseodensification drilling in conjunction with an implant mimicking trabecular bone. Parameters such as BIC and BAFO were measured to quantitively assess the degree of osseointegration and relative bone volume. A sheep model was used as it a highly translational species. Furthermore, the hip was selected due to its low-density bone which would emphasize changes in bone volume more apparent as a function of time.

Although commonly used in research and clinical settings, the conventional drilling protocol can potentially have adverse effect on the implant’s stability in low-density bone. To address the issue of stability, the utilization of grafting materials is an option, which do come with their respective drawbacks. An example, autologous grafts remain the *‘gold’* standard, they are met with donor site morbidity. Other grafts, such as allografts, alloplasts, xenografts, etc., face issues with respect to osteoconduction, osteoinduction, and/or host rejection (24). The osseodensification technique conserves bone fragments (bone chips) that would have otherwise been removed from the osteotomy due to the subtractive nature of conventional drilling (2). These bone chips, which function as autografts, promote osteogenesis in the implant bed which is essential for osseointegration and implant stability (21).

With the exception of the difference observed in BAFO% between OD-CCW and R samples, there were no other significant histomorphometric differences. These results elude that the TM design did not exert any influence with respect to osseointegration within the trabecular network. The higher BAFO% measured in the OD-CCW samples can be linked to presence of bone chips “trapped” within the trabecular network portion of the implant, which were absent in samples drilled via the R protocol. In addition, the increased BAFO% observed in OD-CCW can be a direct result of these bone chips acting as nucleating sites for osteogenesis during the healing period, thus contributing to increased bone volume over time.

As the data shows that the TM did not strongly influence osseointegration and bone volume, and the osteogenesis observed may be correlated to the osteoconductive nature of the autologous bone chips found within the porous network as well in proximity to the implant surface. Osteocytes typically release chemical factors such as nitric oxide (NO), prostaglandin E2 (PGE2), and adenosine triphosphate (ATP), all of which activate bone formation (25). Differentiation of mesenchymal stem cells into osteoblasts is also influenced by certain growth factors such as bone morphogenic protein (26).

Future studies comprising longer time points *in vivo* are suggested so that the extent of bone formation and osseointegration could be better observed.
